# Enhancement of alkaline conductivity and chemical stability of quaternized poly(2,6-dimethyl-1,4-phenylene oxide) alkaline electrolyte membrane by mild temperature benzyl bromination

**DOI:** 10.1039/d0ra06852g

**Published:** 2020-10-06

**Authors:** Murli Manohar, Dukjoon Kim

**Affiliations:** School of Chemical Engineering, Sungkyunkwan University Suwon Kyunggi 16419 Republic of Korea djkim@skku.edu

## Abstract

A series of quaternized polyphenylene oxide (QPPO) based alkaline electrolyte membranes with different degrees of quaternization were synthesized *via* a benzyl bromination method at mild temperature (75 °C). Quite a high hydroxide conductivity under the reduced water uptake and swelling was exhibited by this method. When the degree of bromination measured from ^1^H NMR analysis was 30%, the corresponding hydroxide ion conductivity was 0.021 S cm^−1^. The chemical stability of the QPPO membranes was excellent, showing only 3% weight loss in 3 M NaOH solution during 1 month. The fuel cell performance test under H_2_/O_2_ exhibited the power density of 77 mW cm^−2^ and the current density of 190 mA cm^−2^ at 70 °C. Such excellent properties of QPPO membranes resulted from the achievement of the quaternization at the benzyl position, specifically.

## Abstract 2

A series of quaternized polyphenylene oxide (QPPO) based alkaline electrolyte membranes with different degrees of quaternization were synthesized *via* a benzyl bromination method at mild temperature (75 °C).

## Graphical abstract

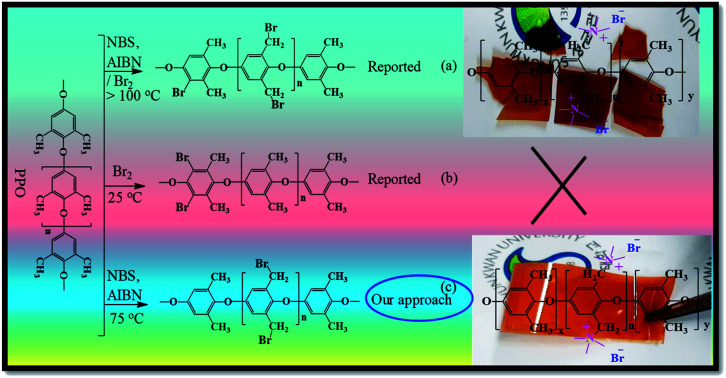

## Introduction

While a proton exchange membrane fuel cell (PEMFC) allows only noble metal catalysts such as platinum (Pt) in the strong acidic environment for its successful operation, much cheaper metal catalysts can still be used in anion exchange membrane fuel cells (AEMFCs).^1,2^ In association with such an advantageous characteristic of AEMFCs over PEMFCs.^3,4^ there have been numerous studies on the development of alkaline electrolyte membranes (AEMs).^5–11^

Even though several types of AEMs have already been commercialized, there are still some criticisms against them in association with the poor chemical and mechanical stability along with low alkaline conductivity. It is noticed that those cons are mostly caused by the nature and location of ion conductive groups such as quaternized ammonium in their synthesis. Due to the high amount of quaternary ammonium groups in the polymeric matrix, the high polar–polar interaction between the quaternary ammonium and water molecules and β-hydrogen elimination^12–15^ hugely affect the structural change in polymer membranes. While the high polar–polar interaction leads to high water uptake or sometimes dissolution of polymer electrolyte, the β-hydrogen elimination (Hofmann or E2), direct nucleophilic substitution (S_N_2), and ylide formation reactions cause the degradation of polymer backbone structure.

The quaternized ammonium is usually provided after the generation of brominated sites. Chloromethyl methyl ether (CMME) was frequently used for quaternization, but as CMME shows carcinogenic and harmful effect on human health,^16^ tin chloride and trimethylchlorosilane have been recently replaced for it in the preparation of AEM and other dual-layer/bipolar membranes.^17–20^ In fuel cell, many hydrocarbon-based membranes are generally prepared from aromatic polymers in order to sustain their inherent thermal, mechanical, chemical resistance against strong acidic and basic conditions. Bromine water and *N*-bromosuccinimide (NBS) have been often used as bromination agents for aromatic polymers.^21–23^ Sisto *et al.* and Tongwen Xu *et al.* reported aryl bromination at room temperature,^24,25^ while some reported benzyl bromination with bromine water and NBS at elevated temperature.^25–27^ As the bromine water generates a great deal of exothermic heat, the polymer backbones are often decomposed during the bromination reaction.^28^ Another weakness of bromine water is that it usually leads to the bromination at the aryl position of the polymer backbone. When the polymers are brominated at aryl position, the quaternization is not easily established because it does not follow the Menshutkin reaction. As a result, it decreases the number of ionic functional sites in polymer and thus low hydrophilicity and low ion conductivity.^25,29^ Being different from bromine water, NBS is reported to possibly generate bromine radicals at benzyl position. When the aromatic polymers are brominated at this benzylic position, the quaternization is feasibly established and thus it increases the hydrophilicity and conductivity.^25^ However, as the conventional bromination condition using NBS requires high temperature around 130–140 °C, there were still some problems associated with the generation of high exothermic heat and in part production of aryl bromination.

As the intake of water in membrane matrix to enhance ion transport is likewise quite important to attain high AEMFC performance,^13^ an appropriate amount of water in quaternized membrane in AEMFC operation is required for optimal cell operation.^14,15^ In consideration of this water uptake, this aryl quaternization is not quite preferable in establishment of large ion clusters to accommodate enough water, because the direct ion conduction group attached on the rigid rod aromatic backbones are hard to aggregate together. Therefore, the present work has been focused on benzylic bromination at mild temperature to avoid the exothermic heat generation and polymer burning. To be suitable for electrochemical applications, the establishment of enhanced conductivity along with the reduced swelling of the flexible membrane with high degree of bromination is explored.

Poly(2,6-dimethyl phenylene oxide) (PPO) is one of the most important classes of the aromatic polymers containing aryl and benzyl groups. Nowadays as a base material, PPO has been extensively studied for their versatile electrochemical applications such as electro-dialysis, fuel cells, batteries, diffusion dialysis, electrolysis and electro deionization due to its easily modifiable structure with robust stability.^5–11^ The present work reflects the characterization of a new class of PPO based alkaline membrane synthesized at the mild bromination temperature using NBS. This bromination process is expected to improve not only the chemical and mechanical stability of the membrane but also its IEC and conductivity along with controllable water uptake. The effect of benzyl bromination degree was in-detail investigated on the physical and electrochemical properties of membranes related with the H_2_/O_2_ single cell performance.

## Experimental

### Chemicals

Poly(2,6-dimethyl-1,4-phenylene oxide) (PPO) was purchased from Sigma-Aldrich (St. Louis, USA). *N*-Bromosuccinimide (NBS, 98.0%) and azobisisobutyronitrile (AIBN, 98.0%) were purchased from TCI (Japan). Potassium hydroxide (KOH, 85%), chlorobenzene (99.5%), isopropyl alcohol (IPA, 99.5%), methanol (99.5%), *N*-methyl pyrrolidone (NMP, 99.5%), and tri-methyl amine (TMA, 99.0%) were purchased from Daejung Reagents & Chemicals (Korea).

### Preparation of electrolyte membrane

In this scheme, the intended bromination site was the aliphatic position of PPO. The degree of bromination was optimized by application of suitable experimental conditions such as amount of reagents, reaction temperature and time. PPO was dissolved in chlorobenzene in a 3-necked round bottom flask under continuous stirring for 1 h. NBS and AIBN were added to proceed the reaction for 3–9 h at 75 °C to prepare the brominated PPO (BPPO) with different degrees of bromination. The reaction mass was cooled down to room temperature and then precipitated in 2-propanol/methanol mixture. After the BPPO product was washed several times with methanol to remove unreacted catalyst and other impurities, it was dried at room temperature and then packed under N_2_ atmosphere. After TMA was added to BPPO solution, the quaternization reaction was conducted at 80–85 °C for 10 h. The quaternized PPO (QPPO) solution was cast on the clean glass plate and then dried in a vacuum oven at 55–60 °C for 24 h to prepare membranes. The prepared membranes were equilibrated in 1.0 M NaOH solution and then washed with deionized water several times. The degree of bromination was controlled by bromination reaction time (3, 6, and 9 h) and the resulting membranes are accordingly designated as QPPO-1, QPPO-2, and QPPO-3.

### Membrane characterizations

The chemical structure of the synthesized BPPO and QPPO were analyzed using ^1^H-NMR and FTIR, TGA, and FE-SEM. ^1^H NMR characterized with (NMR, Unity Inova) equipped with a 500 MHz high-resolution NMR console (S/N: S010002), a 51 mm Bore Oxford super conduction magnet (S/N: 70418) with using chloroform (CDCl_3_) as solvent. Fourier transform infrared spectroscopy (FT-IR, Bruker, Germany) has been used for the functional group investigation with IR frequency range from 4000 to 600 cm^−1^. Thermogravimetric analysis performed with thermogravimetric analyser (TGA, TG7300, SEICO INST) for the thermal stability of the prepared membranes. FE-SEM analysis has been tested for different membranes (surface, morphology, cross-section) with JEOL, JSM 7000 F, Japan at 15.00 kV. The tensile properties of the membrane were measured using a universal tensile machine (UTM model 5565, Lloyd, Fareham, UK) with a 250 N load cell at room temperature.

The ion-exchange capacity (IEC) was determined by the classical titration method. The dry membrane was equilibrated in 1.0 M NaCl solution to convert all ionic sites into Cl^−^ form. After treated with 0.1 M Na_2_SO_4_ solution for 24 h, the membranes were titrated with 0.01 M AgNO_3_ solution using phenolphthalein indicator. IEC (meq. g^−1^) was obtained from the following equation:1
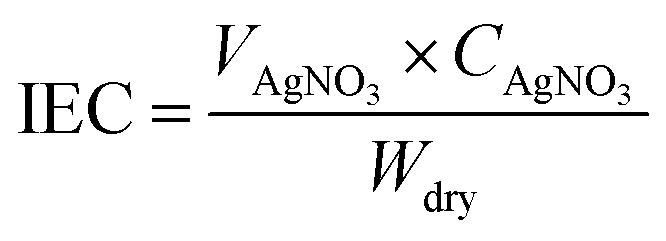


### Water uptake and chemical stability

Water uptake was determined by measuring the weights of the dry and hydrated membrane samples. First, dry the membrane (under vacuum at 60 °C for 10 h), and noted the weight of the dry membrane (*W*_d_). Later, membrane was immersed into deionized water for 24 h at 25 °C, and weight of wet membrane (*W*_w_) was noted after the removal of surface water by absorbing dry paper. The water uptake (WU) was estimated by following eqn (2):2
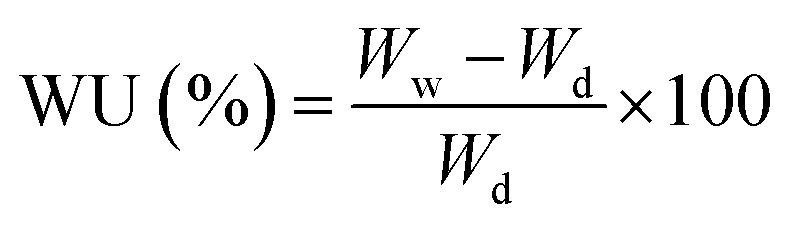


Alkaline stability was estimated by measuring the weight, IEC, conductivity loss of the sample (membrane (2 × 2 cm^2^)) before and after immersion in 3 M NaOH solution at 25 °C for a week.

### Ion conductivity

In-plane hydroxide ion conductivity was measured for all membranes using the four-electrode alternating current impedance spectroscopy (IM6eX impedance unit, Zahner Elektrik, Germany) accompanied with BEKKTECH cells (USA) under 5 mV with four platinum (Pt) electrodes. AC frequency range was from 1 MHz to 1 Hz at 95% relative humidity (RH). Before its measurement, the membrane was immersed in KOH solution (1 M) for at least 2 days for complete ion exchanges into OH^−^ form, followed by several times washing with DI water. The sample dimension was 1 cm × 3 cm × ∼70 μm, and the conductivity (*σ*) of membrane was measured from eqn (3).3
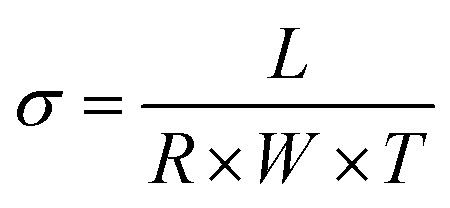
Here, *R* is the ohmic resistance, and *L* (=0.425 cm) is the distance between the anode and cathode electrodes. *W* is the width, and *T* is the thickness of the membrane.

### Fuel cell performance

The membrane electrode assembly (MEA) was firstly fabricated for fuel cell performance test. The catalyst ink was prepared by mixing 0.1 g of Pt/C (40%), 1 mL of DI water, 0.66 g of Nafion ionomer (5 wt% in IPA), and 8.042 g of IPA. After the mixture was sonicated using a sonicator (Sonomasher, SL Science, Korea) for 30 min, the solution was sprayed onto a gas diffusion layer (GDL) using a sprayer gun (Model GP-1, Japan 21701) in 5–10 min intervals. The MEA was prepared by pressing the catalyst coated membrane using a heating press (Ocean Science, Korea) at 105 °C and 5 MPa for 3 min. The active area of MEA for this process was 6.25 cm^2^ and the Pt loading amount of both anode and cathode was 0.35 mg cm^−2^ each. The H_2_/O_2_ fuel cell was operated at 70 °C under 90% relative humidity (RH). The polarization curve for MEA fabricated with each membrane was obtained using a unit cell station (SPPSN-300) provided by CNL Energy (Korea) at 300 cm^3^ min^−1^ H_2_ and O_2_ flow rate.

## Results and discussion

### Synthesis of QPPO

**Fig. 1:**
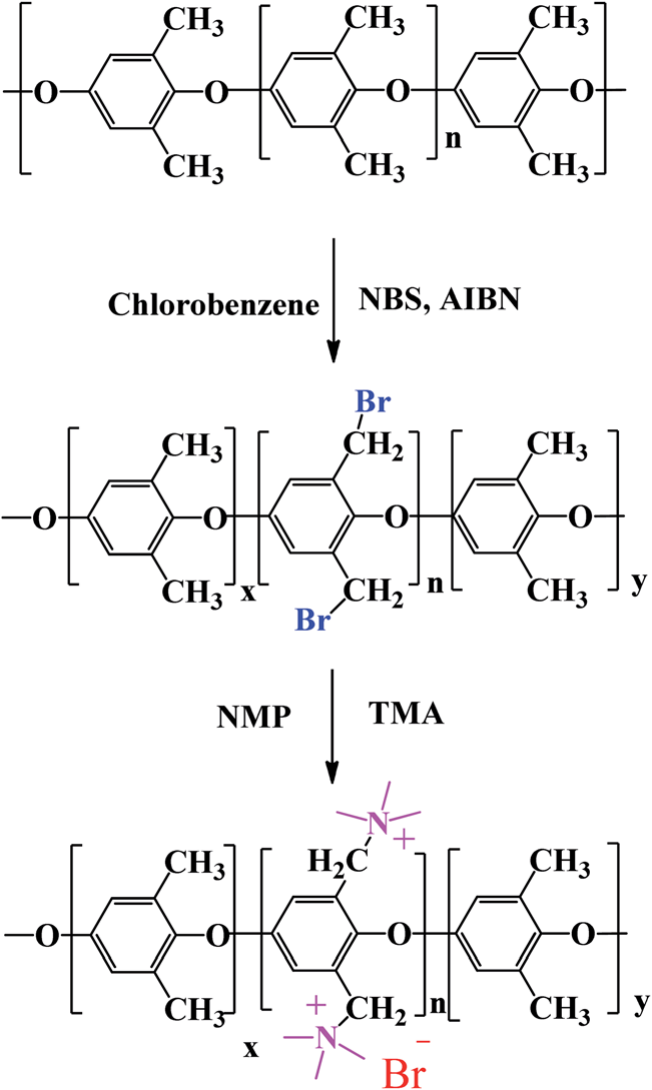
Synthesis of quaternized PPO (Q-PPO).

**Fig. 2:**
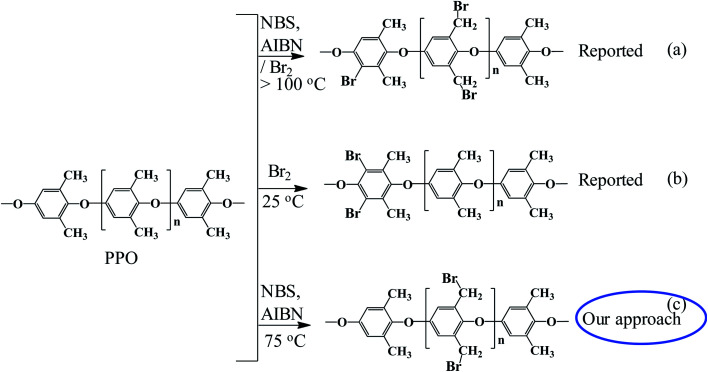
Comparison of the proposed synthetic scheme of Br-PPO with the reported ones.

Our approach was to brominate the PPO at the benzyl position. As the bromine water brominate mostly the aryl position of the polymer and release lots of exothermic heat, we used NBS instead in this study. Being different from the high temperature bromination above 130 °C,^21–27^ this NBS based bromine substitution was more selective at benzylic position at 75 °C.^25–33^ as shown in the synthetic scheme in Fig. 1. Fig. 2 shows the comparison between the reported and recent approached of BPPO.

**Fig. 3:**
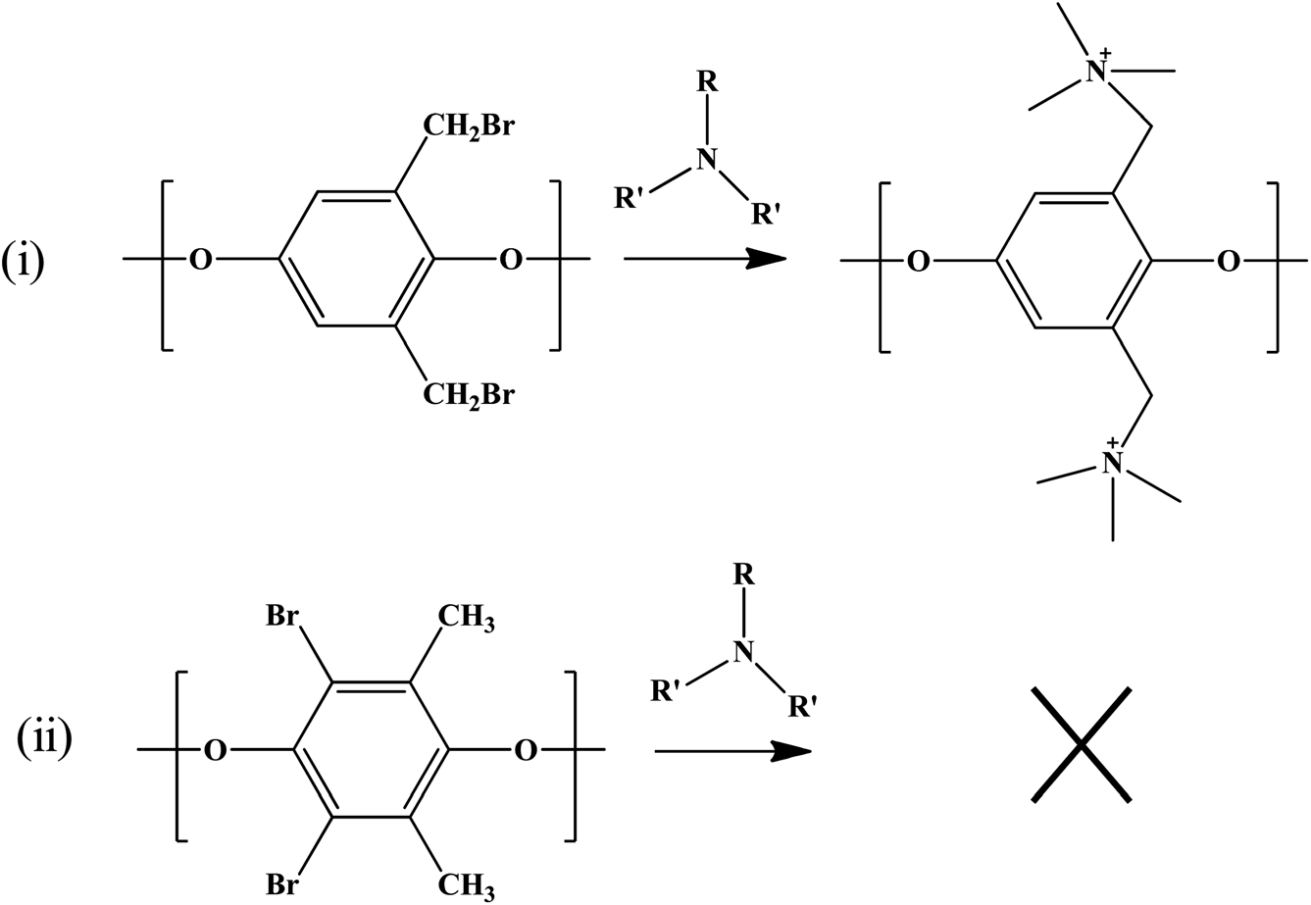
Menshutkin reaction for the (i) benzyl and (ii) aryl brominated substituted bromine.

QPPO was successively synthesized from BPPO by quaternization of its brominated sites applying TMA. As the aliphatic amine groups are highly reactive with bromomethyl groups, the BPPO with higher DOB could accommodate more amount of amine groups during quaternization reaction. The conversion of bromobenzyl methyl into quaternary ammonium follows Menshutkin reaction mechanism shown in Fig. 3. Menshutkin reaction^17,18^ proceeds only at the alkyl and benzyl positions to form quaternary ammonium salts as shown in Fig. 3(i), not at the aryl substituted bromine (Fig. 3(ii)). Therefore, the bromination at the aryl position will increase the stiffness of the polymer electrolyte membrane with the increase of hydrophobicity.^25^

**Fig. 4:**
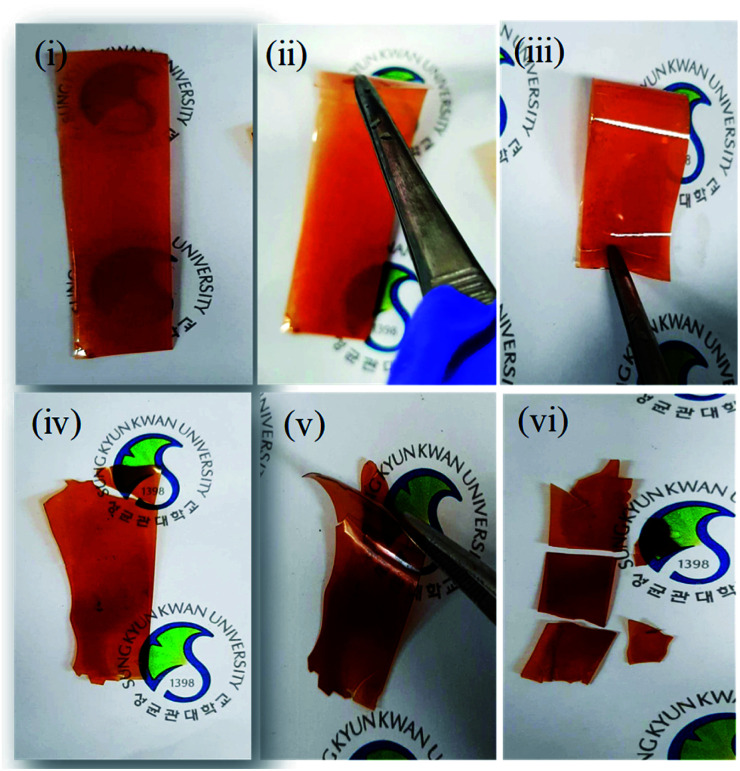
Optical images of the prepared membranes brominated at (i, ii and iii) benzylic and (iv, v and vi) aromatic positions.

In association with this mechanical stiffness and hydrophobicity, the bromination at aliphatic and aromatic position resulted in different mechanical properties. When the bromination occurs at the benzylic position, however, the membrane was quite flexible, and thus no mechanical failure was observed even after many repeated folding processes (Fig. 4(i–iii)). As the membrane synthesized after bromination at aromatic position became so brittle that it was easily broken under repeated folding test (Fig. 4(iv–vi)).

### Characterizations

**Fig. 5:**
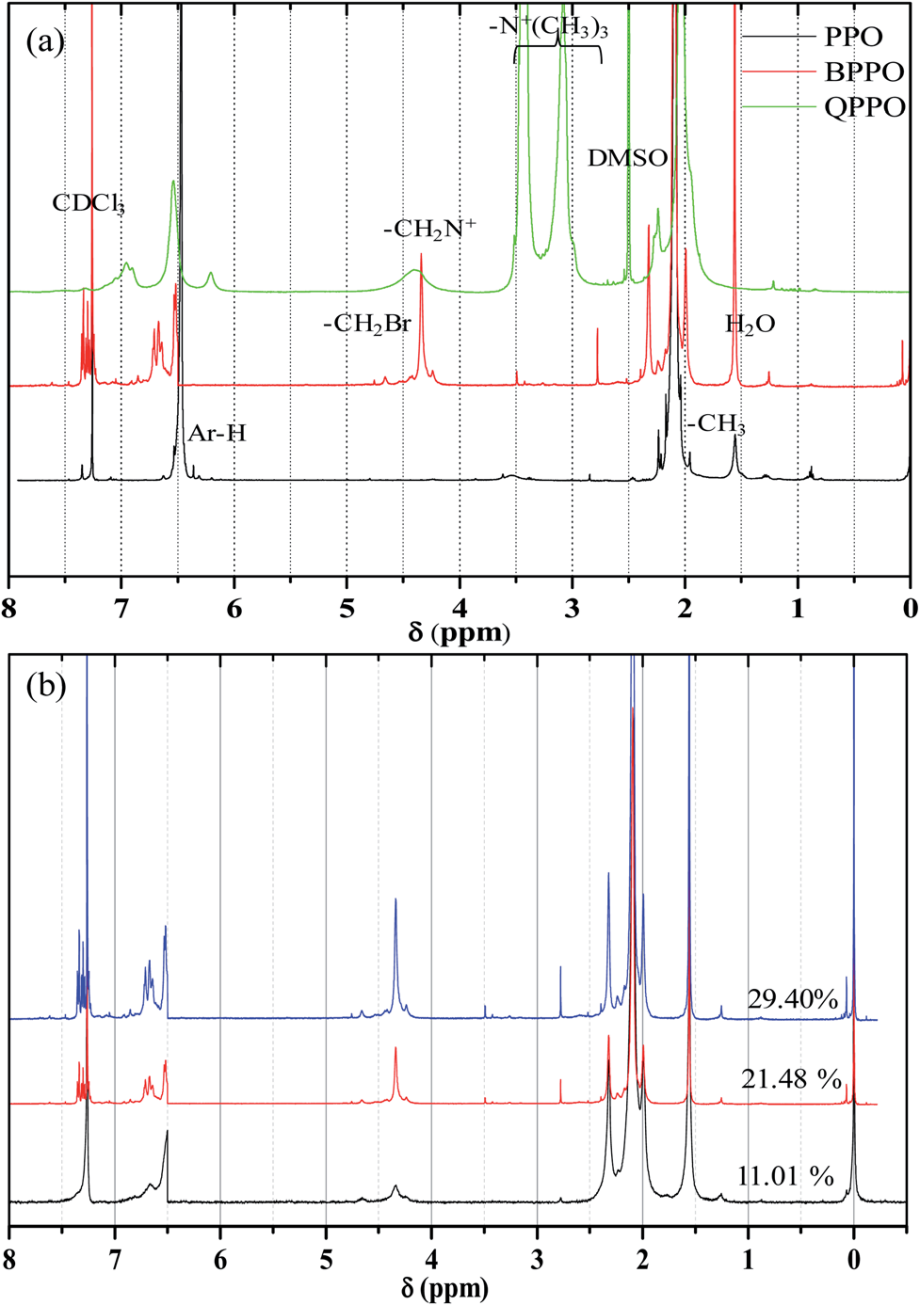
(a) ^1^H NMR spectra for (a) PPO, Br-PPO, and Q-PPO and (b) Br-PPO with different degree of bromination.

^1^H NMR spectra of PPO, BPPO, and QPPO are shown in Fig. 5. For the PPO spectrum, the aromatic protons (Ar-H) and aliphatic protons (–CH_3_) were observed at 6.50 ppm and 2.00 ppm, respectively. After bromination at benzyl position, the new characteristic proton signal from –CH_2_Br appeared at 4.33 ppm and multiplets were observed at 6.5–6.7 ppm from the shift of Ar-H. After quaterization (QPPO), Ar-H signals were shifted toward 6.50–7.20 ppm and aliphatic –CH_3_ signals were also from 1.70–2.00 ppm to 3.00–3.40 ppm (Fig. 5a). The formation of –CH_2_–N^+^ shows the broad signal at 4.00–4.60 ppm. The DOB was controlled by the feed ratio of NBS to initiator and determined from ^1^H NMR spectrum shown in Fig. 5b. When the bromination was conducted for 3 h, a new NMR signal with low intensity was observed from –CH_2_Br at 4.33 ppm without any signal occurrence at 6 ppm, indicating that the bromination begins only at the benzyl position of PPO. When the bromination takes place at the aryl position, the NMR signal was reported to appear at 6.00 ppm rather than at 6.40 ppm associated.^25^ When the reaction time reaches up to 6 h and 9 h, the signal intensity at 4.33 ppm increases, implying the high concentration of –CH_2_Br. The DOB was calculated from the eqn (4).^34^ It increased with increasing reaction time.4
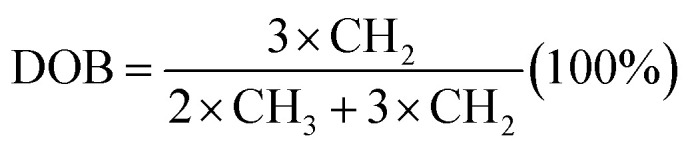
where CH_2_ and CH_3_ are the proton signal intensity of the aliphatic methyl and bromo-methyl groups, respectively.

**Fig. 6:**
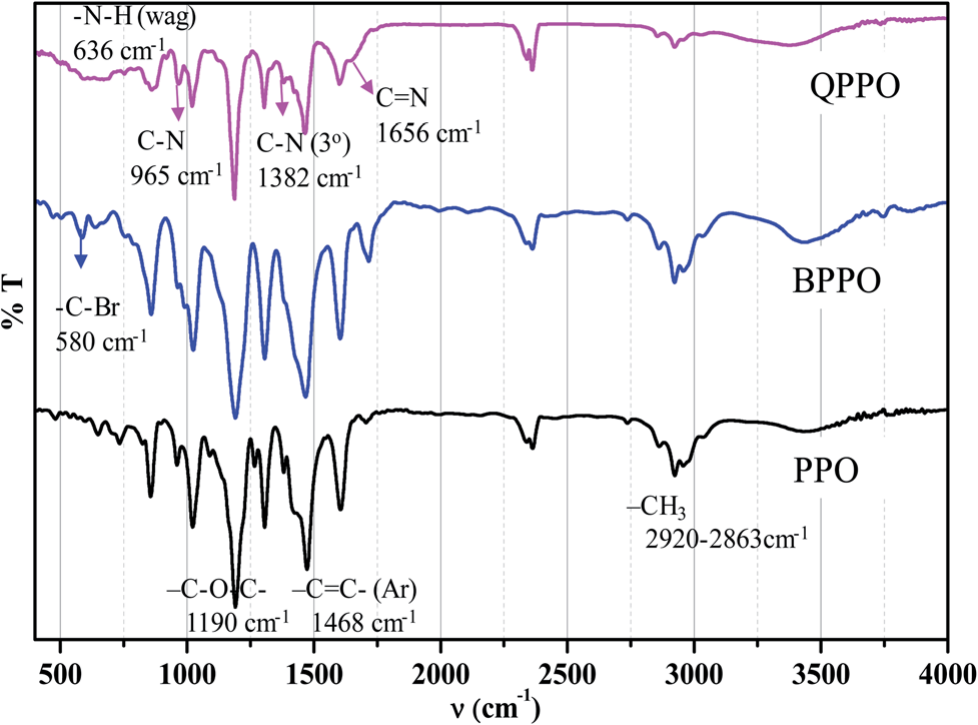
FT-IR spectra for PPO, Br-PPO and Q-PPO.

**Fig. 7:**
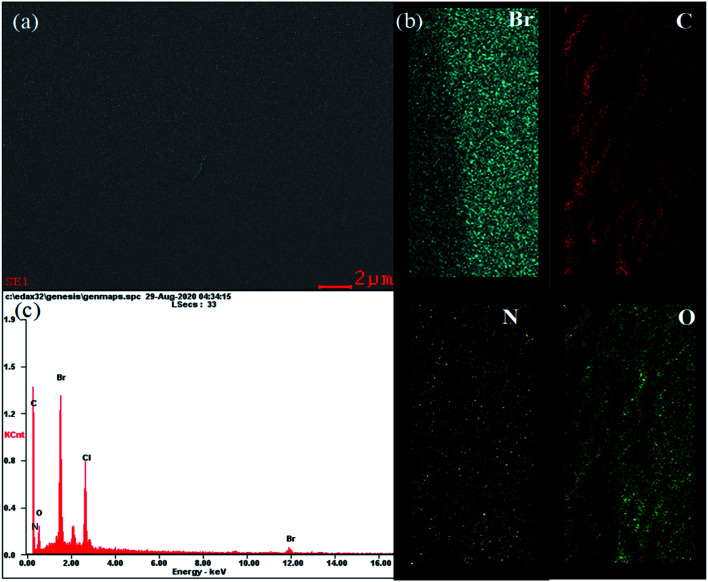
(a) FE-SEM images; (b) elemental mapping images for Br, C, N and O; and (c) EDS spectrum of QPPO-3 membrane.

The FTIR-ATR spectra of the BPPO and QPPO are shown in Fig. 6. The aromatic C–H stretching was observed at 1600 and 1473 cm^−1^, and –CH_3_ at 2920 and 2863 cm^−1^, –C–O–C– at 1190 cm^−1^, and –C=C– at 1768 cm^−1^ are observed from PPO backbone. The production of bromobenzyl group was confirmed from the IR band at 580 cm^−1^ of –C–Br in BPPO, and that of quaternization was from a broad band of –C=N at 1656 cm^−1^. There are no cracks and holes in the membrane surface (QPPO-3) as shown in Fig. 7a. The EDS mapping images of Br, C, N and O elements composing the QPPO-3 membrane are represented in Fig. 7b along with the corresponding EDS spectrum in Fig. 7c. The homogeneous distribution of these elements, especially of Br, is clearly proved by EDS mapping analysis, which confirms the successful synthesis of BPPO and successful preparation of the corresponding membrane.

**Fig. 8:**
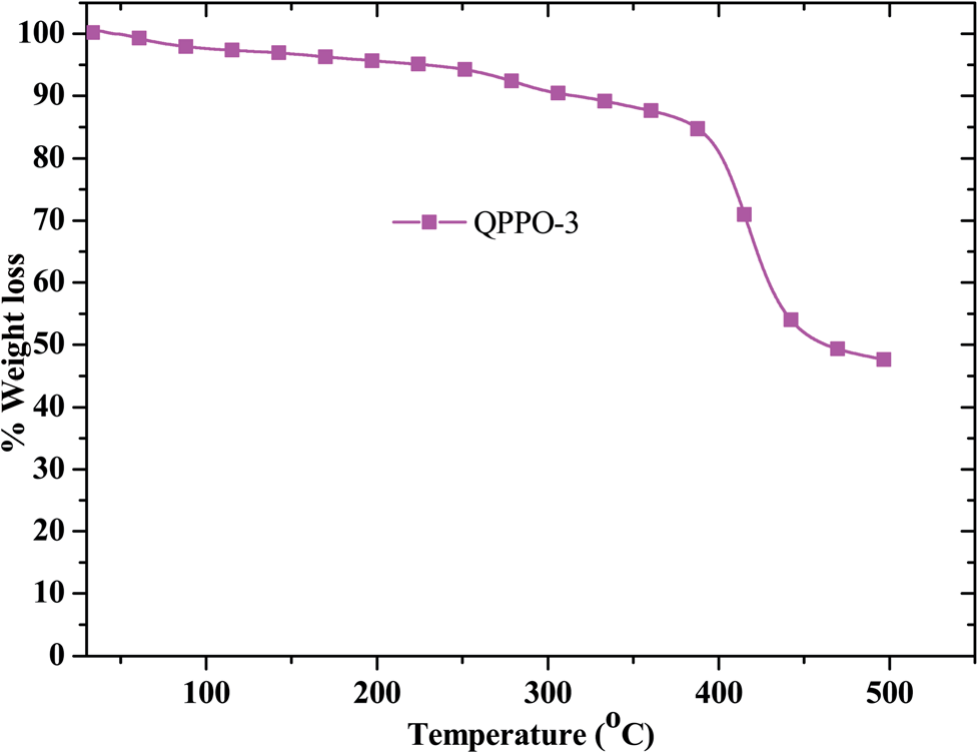
TGA behavior for QPPO-3.

**Fig. 9:**
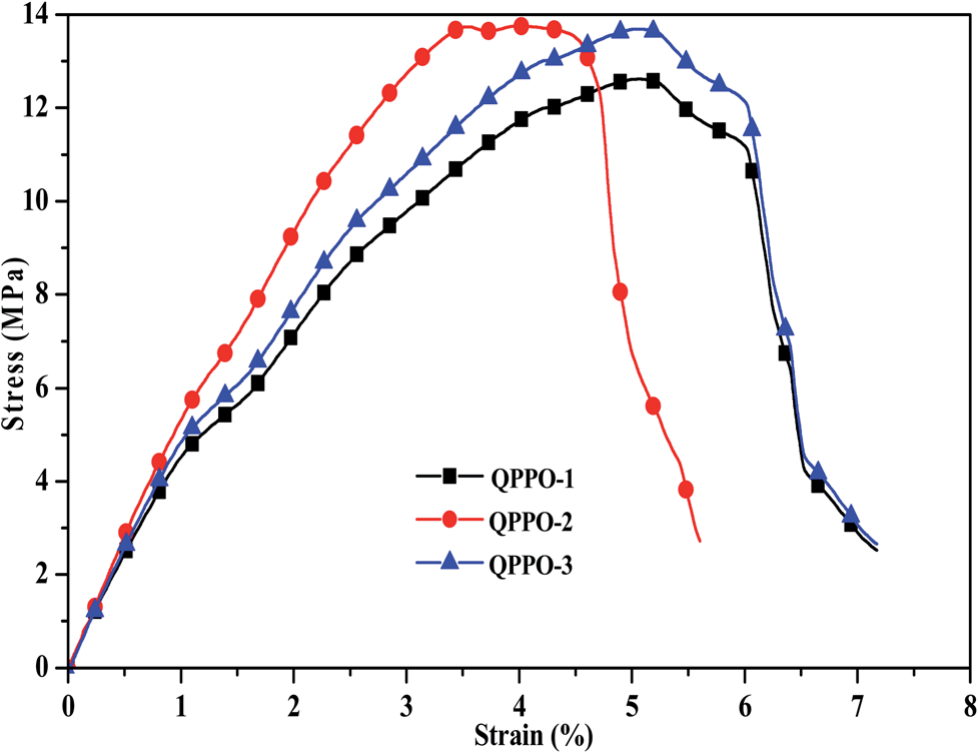
Stress–strain behavior for QPPOs membranes.

As the fuel cell performance was performed above 80 °C for long period time, it requires thermal stability analysis. The thermal stability was examined for QPPO-3 up to 500 °C and the resulting TGA behavior is presented in Fig. 8. While QPPO-3 membrane was quite stable up to 200 °C, the ionic functional groups were degraded from this temperature. Mechanical property is also an important aspect for the application of polymer electrolyte membrane. High tensile strength with good flexibility of the membrane is suitable for the feasible and sustainable fabrication of membrane electrode assembly. The stress–strain behavior of the membranes exhibited that QPPO-2 showed the highest tensile strength of 13.74 MPa with a breaking strain of 5.59%, while QPPO-3 showed the tensile strength of 13.69 MPa with a breaking strain 7.16% (Fig. 9).

### Hydroxide ion conductivity

**Fig. 10:**
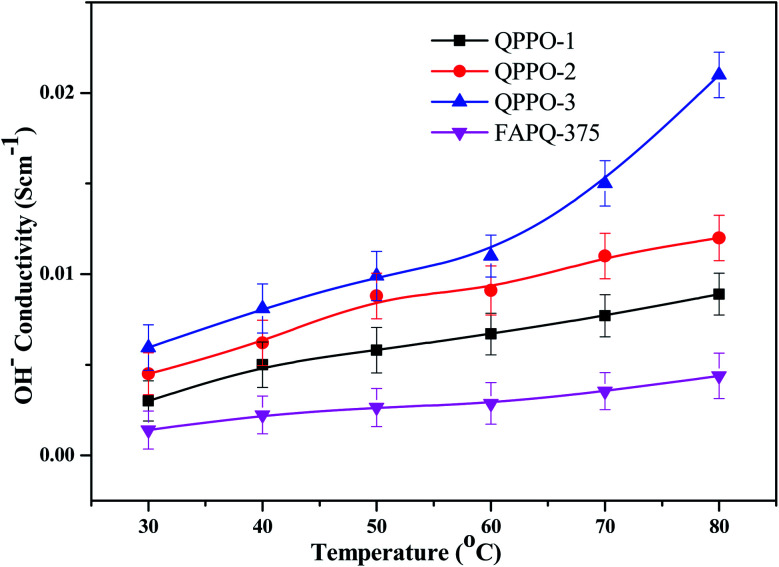
OH^−^ conductivity for QPPOs alkaline membranes and FAPQ-375 commercial membrane.

The ion conductivity depends on the type and number of ion transferrable functional groups in the polymer membrane, as these functional groups influence the size and shape of the hydrophilic ionic domains through which hydroxide ion transports. The hydroxide ion conductivity of the prepared QPPO membranes is presented in Fig. 10 in comparison with that of the commercial one. QPPO-3 possesses more quaternary ammonium groups than QPPO-1 and QPPO-2, QPPO-3 shows the highest conductivity of 0.021 S cm^−1^, while the commercial FAPQ-375 PP shows 0.0045 S cm^−1^ at 80 °C.

### Alkaline stability

**Fig. 11:**
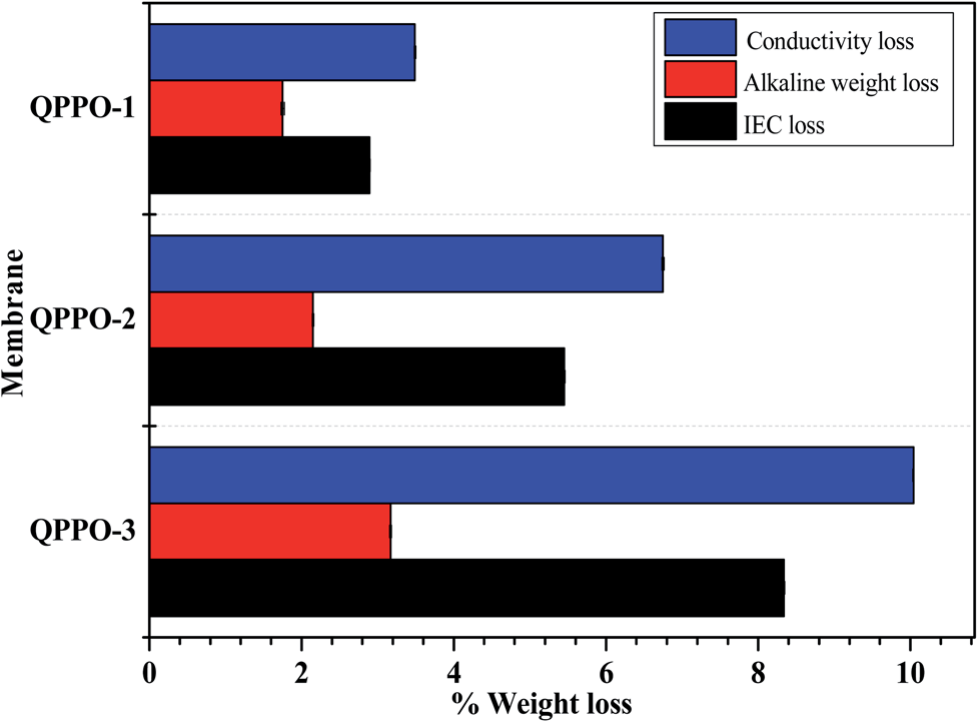
Alkaline stability test in terms of loss% of weight, conductivity, and IEC for QPPOs alkaline membranes.

The quaternary ammonium group is reported to be unstable in high pH solution, due to the degradation *via* Hofmann elimination (E2), ylide formation and nucleophilic substitution (S_N_2).^12,35,36^ The alkaline stability of the prepared QPPO membranes were tested in high concentrated alkaline solution. As shown in Fig. 11, the weight loss of QPPO-3 membrane in acidic and alkaline condition was 2.85% and 3.25%, respectively. QPPO-1 and QPPO-2 showed lower weight loss than QPPO-3 due to the lower quaternary ammonium concentration. The loss% of IEC and conductivity was also measured for the alkaline stability test of the membranes. In Fig. 11, QPP-3 showed the highest IEC and conductivity loss% of 8.34% and 10.04% respectively, while QPPO-1 showed quite low loss% of IEC (2.89%) and conductivity (3.49%), for the same reason as the weight loss.

### Water uptake and swelling ratio

**Fig. 12:**
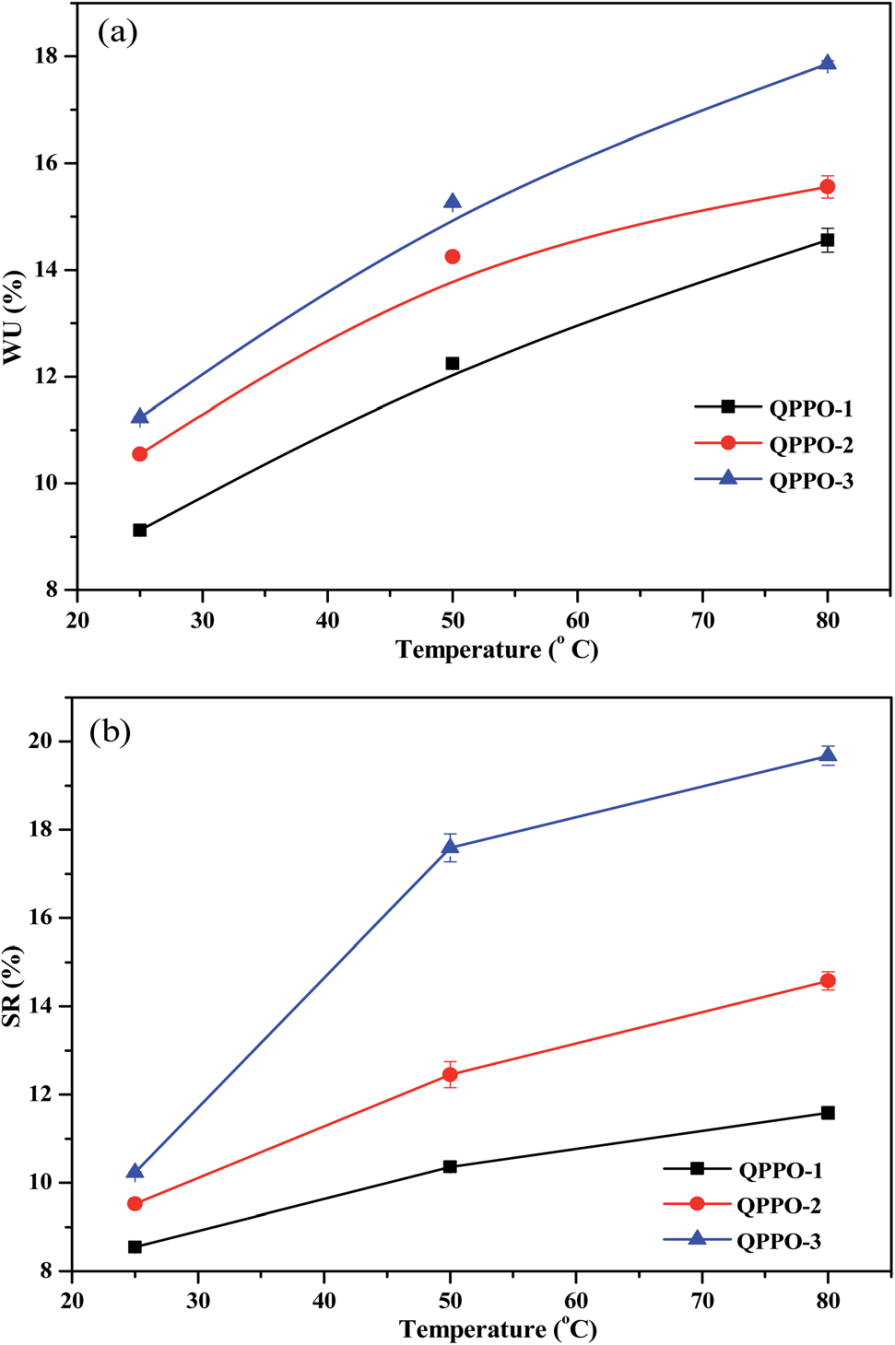
(a) Water uptake and (b) swelling ratio for QPPOs alkaline anion membranes.

The water uptake (WU) in the polymer membrane defines the hydrophilic domain size and structure, and thus it is pre-requisite for the hydroxide ion transport. The swelling ratio of the membrane is also important property to be investigated as it has significant effect on interfacial stability between membrane and catalyst. As shown in Fig. 12a and b, QPPO-3 shows the highest WU and swelling ratio as the degree of bromination of QPPO-3 was the highest among three membranes. The presented work shows the highest WU of 18% and swelling ratio of 19% for QPPO-3 and the lowest WU of 11% and swelling ratio of 10% for QPPO-1 at 80 °C. It was quite surprising that QPPO-3, a type of hydrocarbon based polymer membrane shows such a high the hydroxide ion conductivity of 0.0052 S cm^−1^ (IEC of 1.83 meq. g^−1^) at such a low water uptake of 18% even at 80 °C. For the better comparison among different types of hydrocarbon based polymer membranes, the hydroxide conductivity and IEC along with water uptake, swelling ratio are included in Table 1.^5,23,37–40^ QPPO-3 membranes illustrated much higher conductivity and IEC than QPPO-1 and QPPO-2. QPPO-1 with IEC = 1.23 meq. g^−1^ exhibited the OH^−^ conductivity of 0.002 S cm^−1^, which was lower than that of QPPO-2 with IEC = 1.51 meq. g^−1^, 0.0044 S cm^−1^ and QPPO-3 with IEC = 1.83 meq. g^−1^, 0.0052 S cm^−1^.

**Table 1:** Water uptake (%), ion-exchange capacity (IEC) and swelling ratio (%) for prepared with reported membranes

Membrane	WU (%)	IEC (meq. g^−1^)	SW (%)	Ref.
PPO-TMA-20	25.9	1.39	7.4	23
GT64-20	43	3.16	—	37
PPO-TMA^+^Cl^−^	104 ± 4	2.1 ± 0.1		38
30PPOFC6NC6	57 ± 3	1.60	16	5
X60Y15	28	2.76	6	39
QAPPO-40	72.2	2.27	∼10	40
QPPO-3	11.25	1.83	7.70	This work

### Fuel cell performance

**Fig. 13:**
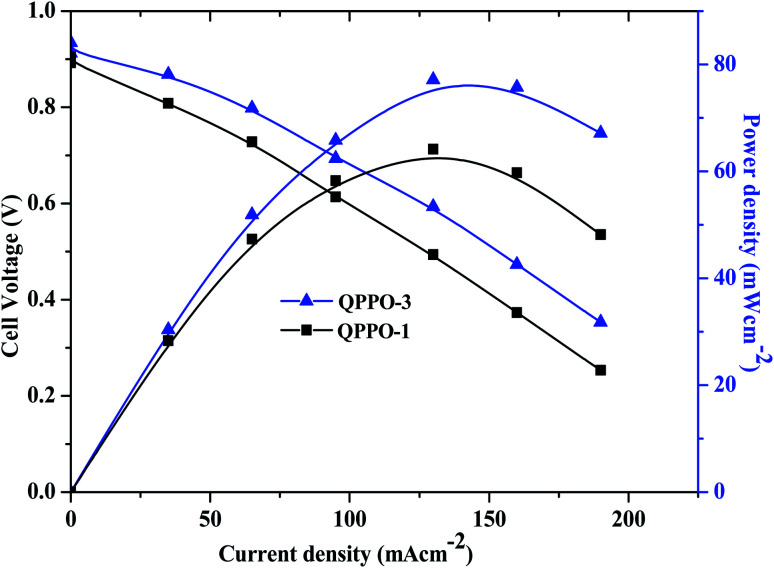
Polarization curves of QPPOs membrane in H_2_/O_2_ single cell AEMFC tests at 70 °C (Pt/C anodes and cathodes; metal loadings 0.4 mg cm^−2^).

The H_2_/O_2_ fuel cell performance of QPPO membranes is shown in Fig. 13. As shown in Fig. 13, the alkaline fuel cell exhibited the open circuit voltage (OCV) of 0.95 V for QPPO-3. QPPO-3 showed a peak power density of 77 mW cm^−2^ at a current density of 130 mA cm^−2^, while QPPO-1 showed a peak power density of 62 mW cm^−2^ at a current density of 130 mA cm^−2^. The comparison of fuel cell performance with other reported works based on brominated and quaternized PPO membranes is shown in Table 2.^41–45^ These all reported works based on the brominated PPO have been investigated for the fuel cell performance obtained using the same catalyst. While M − 5 shows the maximum peak power density of 30 mW cm^−2^, our work shows the maximum peak power density of 77 mW cm^−2^ from QPPO-3 at the same loading amount of the same catalyst. C16D40 shows the maximum peak power density similar to the present work but their metal loading amount was higher than ours.

**Table 2:** Comparisons of fuel cell performance of reported QPPOs

Membrane	Peak power density (mW cm^−2^)	Catalyst		Metal loading		Ref.
		Cathode	Anode	Cathode	Anode (mg cm^−2^)	
M-5	30	Pt/C	Pt/C	0.4	0.4	41
Membrane C	32	Pt/C	Pt/C	0.5	0.5	42
PPO-43-Im0.88F0.2	51.1	Pt/C	Pt/C	0.8	0.8	43
C-QPPO-*x*	31	Pt/C	Pt/C	0.5	0.5	44
C6D60	67	Pt/C	Pt/C	0.50–0.60 (both)		45
C16D40	77	Pt/C	Pt/C	0.50–0.60 (both)		45
QPPO-3	77	Pt/C	Pt/C	0.4	0.4	Present work

## Conclusions

The present work reflects the synthesis and properties of chemically stable QPPO membranes with the different degrees of quaternization for AEMFC. The bromination was conducted at around 75 °C to minimize the exothermic heat release but maximize the benzyl bromination up to 30% capable of feasible quarternisation. This synthetic process also avoided the use of carcinogenic and hazardous chemicals (liquid bromine and CMME) during the introducing of –CH_2_Br. The ion conductivity and water uptake were increased with degree of bromination. QPPOs exhibited excellent alkaline/base stabilities, water uptake, and hydroxide ion conductivity. Among a series of membranes, the highest brominated and quaternized membrane QPPO-3, showed quite high hydroxide ion conductivity at low WU and swelling along with excellent chemical stability. These unique characteristics of QPPO-3 membrane led to the excellent fuel cell performance showing 77 mW cm^−2^ peak power density.

## Conflicts of interest

There are no conflicts to declare.

## Supplementary Material
